# The Effect of Chronic Treatment with the Inhibitor of Phosphodiesterase 5 (PDE5), Sildenafil, in Combination with L-DOPA on Asymmetric Behavior and Monoamine Catabolism in the Striatum and Substantia Nigra of Unilaterally 6-OHDA-Lesioned Rats

**DOI:** 10.3390/molecules29184318

**Published:** 2024-09-11

**Authors:** Elżbieta Lorenc-Koci, Kinga Kamińska, Tomasz Lenda, Jolanta Konieczny

**Affiliations:** Maj Institute of Pharmacology, Polish Academy of Sciences, 12 Smętna Street, 31-343 Kraków, Poland; k.kamin@if-pan.krakow.pl (K.K.); lenda@if-pan.krakow.pl (T.L.); koniecz@if-pan.krakow.pl (J.K.)

**Keywords:** inhibitor of phosphodiesterase 5, sildenafil, 6-OHDA rat model of Parkinson’s disease, dopamine and serotonin metabolism, L-DOPA, nitric oxide–soluble guanylyl cyclase–cyclic GMP (NO–sGC–cGMP) signaling pathway, rotational behavior

## Abstract

The use of phosphodiesterase inhibitors in the treatment of Parkinson’s disease is currently widely discussed. The study aimed to investigate the impact of acute and chronic treatment with the phosphodiesterase 5 inhibitor, sildenafil, at low and moderate doses of 2 mg/kg and 6 mg/kg, and L-DOPA (12.5 mg/kg), alone or in combination, on asymmetric behavior and dopamine (DA) and serotonin metabolism in the striatum and substantia nigra of unilaterally 6-OHDA-lesioned rats. Acute administration of sildenafil at both tested doses jointly with L-DOPA significantly increased the number of contralateral rotations during a 2 h measurement compared to L-DOPA alone. The effect of a lower dose of sildenafil combined with L-DOPA was much greater in the second hour of measurement. However, the acute combined administration of a higher dose of sildenafil with L-DOPA resulted in an immediate and much stronger increase in the number of contralateral rotations compared to L-DOPA alone, already visible in the first hour of measurement. Interestingly, the chronic combined administration of 2 mg/kg of sildenafil and L-DOPA significantly reduced the number of contralateral rotations, especially during the first hour of measurement, compared to the long-term treatment with L-DOPA alone. Such an effect was not observed after the long-term combined treatment of a higher dose of sildenafil and L-DOPA compared to L-DOPA alone. The concentration of DA in the ipsilateral striatum and substantia nigra after the last combined chronic dose of sildenafil (2 or 6 mg/kg) and L-DOPA (12.5 mg/kg) was significantly higher than after L-DOPA alone. In spite of much stronger increases in the DA concentration in the ipsilateral striatum and substantia nigra, the number of contralateral rotations was reduced in the group of rats treated with the combination of 2 mg/kg sildenafil and L-DOPA compared to the group receiving L-DOPA alone. Moreover, the combined treatment with a low dose of sildenafil and L-DOPA had an opposite effect on DA catabolism, as assessed by DOPAC/DA and HVA/DA indexes, and these indexes were reduced in the ipsilateral striatum but increased in the contralateral striatum and substantia nigra compared to the treatment with L-DOPA alone. The results of the present study show that the addition of a low dose of a PDE5 inhibitor to the standard L-DOPA therapy differently modulates rotational behavior, the tissue DA concentration and its catabolism in the striatum and substantia nigra.

## 1. Introduction

Parkinson’s disease (PD) is a progressive age-related neurodegenerative disorder whose most distinctive symptoms are motor dysfunctions, such as bradykinesia, muscle rigidity, resting tremors, freezing gait disturbances, and postural instability [1]. Locomotor deficits result predominantly from the loss of the dopamine (DA)-synthesizing neurons located in the substantia nigra pars compacta (SNc) sending their projections to the caudate-putamen (corresponding in rats to the corpus striatum; STR), which ultimately leads to a drastic decline in DA content in both the SN and STR [2,3]. At the molecular level, factors underlying the degenerative process in PD have not been definitively determined, but it is widely accepted that oxidative stress, mitochondrial dysfunction, neuroinflammation, and the accumulation of misfolded α-synuclein proteins play a fundamental role [4,5,6,7].

Currently, DA precursor 3,4-dihydroxy-L-phenylalanine (L-DOPA) is a commonly used drug to improve motor deficits in the vast majority of PD patients. However, other drugs, such as agonists of DA receptors and blockers of monoamine oxidase B (MAO-B) or catechol-O-methyltransferase are also applied [8]. Despite the high effectiveness of L-DOPA (always combined with the inhibitor of aromatic L-amino acid decarboxylase to prevent systemic side effects) in the alleviation of motor deficits, its long-term administration causes motor complications, such as motor fluctuations and dyskinesia [8,9,10]. Furthermore, L-DOPA replacement therapy is unable to stop or reverse the progression of degenerative alterations. Therefore, new forms of PD treatment are constantly being sought to alleviate the negative effects of both these phenomena. Recently, the attention of researchers has focused on examining the effectiveness of phosphodiesterase (PDE) inhibitors in the treatment of PD [11,12,13,14,15].

Overall, dopaminergic signaling in the STR mainly involves the activation of postsynaptic dopamine D1 and D2 receptors located on the striatal GABA-containing medium-sized spiny neurons (MSNs), which form the only output pathways from the STR [16,17,18]. A population of MSNs expressing DA D1 receptors projects to the substantia nigra pars reticulata (SNr) and the internal segment of the globus pallidus (GPi; corresponding in rodents to the entopeduncular nucleus) forming the so-called “direct pathway” that promotes movement. The second population of MSNs expressing DA D2 receptors projects to the external segment of the globus pallidus (GPe; in rodents the pallidum) forming the “indirect pathway” that inhibits movement. To maintain normal motor functions, a precisely regulated balance between the activity of the direct and indirect output pathways of the STR is necessary. The binding of DA to the striatal D1 receptors localized on the direct pathway activates adenylyl cyclase (AC) that facilitates the production of adenosine 3′,5′ cyclic monophosphate (cAMP) [19,20] while the binding of DA to the striatal D2 receptors on the indirect pathway inhibits AC activity and cAMP production. In addition, a growing body of evidence shows that the DA-mediated stimulation of DA D1 receptors located on striatal interneurons containing neuronal nitric oxide synthase (nNOS) increases NO biosynthesis in the STR. In contrast, the indirect action of DA via the stimulation of DA D2 heteroreceptors decreases NO synthesis [21,22,23,24]. NO, as a unique gaseous neurotransmitter and neuromodulator, penetrates cell membranes. It can easily reach soluble guanylyl cyclase (sGC), abundantly present in the dendritic spines of MSNs [25,26], especially because the shafts of these spines receive direct synaptic inputs from NO-producing interneurons [27]. Binding of NO to sGC enhances the activity of this enzyme and may lead to the increase in the concentration of cGMP as a result of the conversion of guanosine 5′-triphosphate (GTP) to guanosine 3′,5′ cyclic monophosphate (cGMP). The above data show that DA modulates the concentrations of both, cAMP and cGMP, and their functional pools are tightly controlled by a precise balance between their synthesis catalyzed by AC and GC, and their degradation regulated by specific cyclic nucleotide phosphodiesterases (PDEs) occurring in the brain [28,29]. Therefore, the final concentration of cGMP depends on the enzymatic activity of PDEs that degrade this nucleotide, especially PDE1B, which is abundant in the striatum and is mainly responsible for the degradation of cGMP [30,31].

The loss of DA neurons in the SN of PD patients and in animal models of this disease, caused by the DA deficit in the STR and SN, leads to significant changes in the concentration of cyclic nucleotides. It has been shown that the unilateral lesion of DA neurons in the SN leads to an increase in cAMP [32,33], but to a decrease in cGMP concentrations, in the ipsilateral STR [30,33]. The unexpected increase in cAMP in the ipsilateral STR was shown to be associated with an increase in basal AC activity [32]. In contrast, a decreased content of cGMP in the ipsilateral STR was associated with the decreased nNOS expression and activity, which probably led to a down-regulation of the NO-sGC pathway, and additionally with the increased expression and activity of PDE1B [30,33]. Chronic L-DOPA treatment regulates cAMP and cGMP concentrations differently in dyskinetic and non-dyskinetic 6-OHDA lesioned rats [33,34]. In non-dyskinetic rats, L-DOPA increased the cAMP level in the ipsi- and contralateral STR, but decreased cGMP below the basal level only in the contralateral STR. In dyskinetic rats, chronic treatment with L-DOPA resulted in a drastic reduction in cAMP and cGMP contents on both sides of the STR [33,34]. Pretreatment with the PDE inhibitor zaprinast, which prevented the breakdown of both cAMP and cGMP, partially reversed the decline in the cyclic nucleotide concentrations and reduced the severity of L-DOPA-induced dyskinesia [33]. The above data indicate the beneficial effects of the combined treatment with L-DOPA and PDE inhibitors on motor disturbances.

Currently, the interest of researchers is predominantly focused on the use of PDE inhibitors specifically degrading both cAMP and cGMP or only cAMP, which, in the experimental models of PD, were administered alone or in combination with L-DOPA [11,13,35,36,37]. However, there is no experimental data regarding the use of selective cGMP-degrading PDE inhibitors, administered alone or in combination with L-DOPA, in the 6-OHDA-induced rat model of this disease.

Sildenafil used in the present study is a selective inhibitor of PDE5, which belongs to a group of PDEs also comprising PDE6 and 9, that specifically degrade cGMP into inactive guanosine 5′-monophosphate (GMP). This drug was initially approved for the treatment of erectile dysfunction but later received secondary approval for the treatment of pulmonary arterial hypertension [38,39]. Recently, numerous attempts have been made to extend the therapeutic effects of sildenafil to metabolic disorders such as type 2 diabetes and obesity, as well as nephropathy and cardiomyopathy [39,40,41].

PDE5 is most abundantly expressed in the smooth muscles, notable in the vasculature, where it regulates NO/cGMP-mediated vascular relaxation [42], but it was also found in other tissues including the lungs, kidneys and brain [40,43]. In the brain, the expression of PDE5 or its mRNA was found in the cortex hippocampus and striatum, but also in the brainstem and spinal cord [44]. Sildenafil, in addition to its vasodilatory effect, has a neurogenic effect and increases the concentration of cGMP in the cortex, hippocampus, and striatum, as well as in other basal ganglia [45,46,47].

The present study aimed to examine the effects of chronic systemic treatment with the PDE5 inhibitor sildenafil, alone or in combination with L-DOPA, on asymmetric behavior and monoamine catabolism in the STR and SN of unilaterally 6-OHDA-lesioned rats.

## 2. Results

### 2.1. The Effect of Sildenafil and L-DOPA on Asymmetric Behavior of Unilaterally 6-OHDA-Lesioned Rats

**Figure 1:**
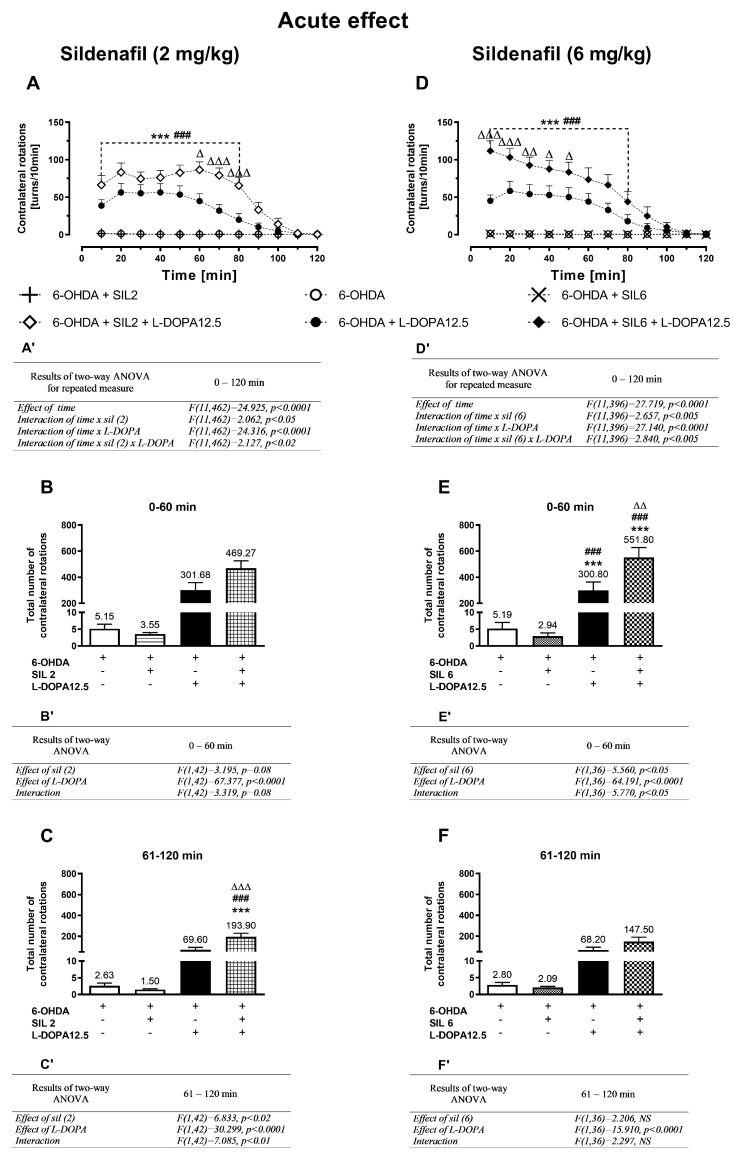
The effects of acute treatment with sildenafil (2 or 6 mg/kg) and L-DOPA (12.5 mg/kg), alone or in combination, on the number of contralateral rotations measured in 10 min intervals for two hours in unilaterally 6-OHDA-lesioned rats (**A**,**D**) and on the total number of these rotations calculated in two periods of 0–60 min (**B**,**E**) and 61–120 min (**C**–**F**). The data are presented as the mean ± SEM; the number of animals in experimental groups is as follows: for 6-OHDA, n = 10; for 6-OHDA+sil2, n = 10; for 6-OHDA+sil6, n = 10; for 6-OHDA+L-DOPA(12.5), n = 12–14; for 6-OHDA+sil2+L-DOPA(12.5), n = 12; for 6-OHDA+sil6+L-DOPA(12.5), n = 10. A two-way ANOVA for repeated measures was used to statistically analyze time-dependent changes in the number of contralateral rotations (**A**,**D**); its effects are shown below as (**A’**,**D’**). Statistical analysis of the data presented in (**B**,**C**,**E**,**F**) was performed using a standard two-way ANOVA; its effects are shown below as (**B’**,**C’**,**E’**,**F’**). The abbreviation NS denotes a nonsignificant effect. Symbols indicate the significance of differences according to the Newman–Keuls post hoc test, *** *p* < 0.001 vs. 6-OHDA+veh-; ^###^ *p* < 0.001 vs. 6-OHDA+sil2- or 6-OHDA+sil6-; ^∆^ *p* < 0.05, ^∆∆^ *p* < 0.01, ^∆∆∆^ *p* < 0.001 vs. 6-OHDA+L-DOPA-treated groups. These symbols were marked only when there was a significant interaction between the studied drugs. Additionally, in (**B**,**C**,**E**,**F**), the mean number of contralateral rotations is given numerically above each bar.

**Figure 2:**
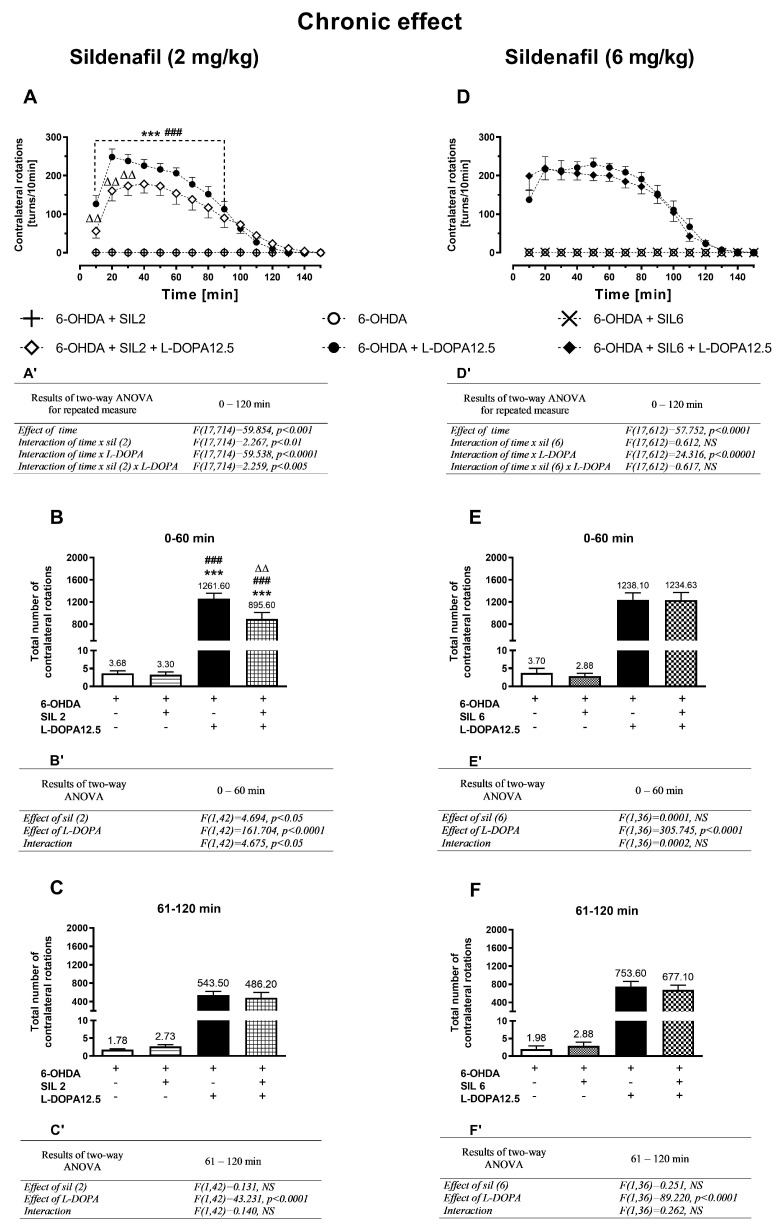
The effects of chronic treatment with sildenafil (2 or 6 mg/kg) and L-DOPA (12.5 mg/kg), alone or in combination, on the number of contralateral rotations measured in 10 min intervals for two hours in unilaterally 6-OHDA-lesioned rats (**A**,**D**) and on the total number of these rotations calculated in two periods of 0–60 min (**B**,**E**) and 61–120 min (**C**–**F**). The data are presented as the mean ± SEM; the number of animals in experimental groups is as follows: for 6-OHDA, n = 10; for 6-OHDA+sil2, n = 10; for 6-OHDA+sil6, n = 10; for 6-OHDA+L-DOPA(12.5), n = 12–14; for 6-OHDA+sil2+L-DOPA(12.5), n = 12; for 6-OHDA+sil6+L-DOPA(12.5), n = 10. A two-way ANOVA for repeated measures was used to statistically analyze time-dependent changes in the number of contralateral rotations (**A**,**D**); its effects are shown below as (**A’**,**D’**). Statistical analysis of the data presented in (**B**,**C**,**E**,**F**) was performed using a standard two-way ANOVA; its effects are shown below as (**B’**,**C’**,**E’**,**F’**). The abbreviation NS denotes a nonsignificant effect. Symbols indicate the significance of differences according to the Newman–Keuls post hoc test, *** *p* < 0.001 vs. 6-OHDA+veh-, ^###^ *p* < 0.001 vs. 6-OHDA+sil2- or 6-OHDA+sil6-, ^∆∆^ *p* < 0.01, vs. 6-OHDA+L-DOPA-treated groups. These symbols were marked only when there was a significant interaction between the studied drugs. Additionally, in (**B**,**C**,**E**,**F**) the mean number of contralateral rotations is given numerically above each bar.

The time-dependent alterations in the intensity of contralateral rotations after single and multiple doses of sildenafil (2 and 6 mg/kg; sil2 or sil6) and L-DOPA (12.5 mg/kg), administered alone or in combination are shown in Figure 1A,D and Figure 2A,D.

#### 2.1.1. Acute Treatment with Sildenafil (2 mg/kg) and L-DOPA (12.5 mg/kg)

Results of two-way ANOVA for repeated measures calculated for the data set presented in Figure 1A, referring to the lower single dose of sildenafil (2 mg/kg; sil2) and L-DOPA (12.5 mg/kg), administered alone or in combination, showed a significant effect of time and interactions: time x sil2 treatment, time x L-DOPA treatment, and time × sil2 × L-DOPA treatment (Figure 1A’). Neither sil2 alone nor the vehicle evoked contralateral rotations, in contrast to L-DOPA, which produced distinct contralateral rotations already visible 10 min after its first dose. Moreover, the acute combined administration of sil2+L-DOPA significantly increased the number of contralateral rotations measured in 10 min intervals during the whole measurement session lasting 120 min compared to the effect of L-DOPA alone (Figure 1A).

However, due to the larger number of rotations after acute combined treatment in the second hour of measurement, the entire session was divided into two periods: 0–60 min and 61–120 min. The total number of rotations in these periods is presented as a bar graph in Figure 1B,C while the results of the two-way ANOVA are given as B’,C’.

Results of the two-way ANOVA calculated for the total number of contralateral rotations during the period of 0–60 min (Figure 1B’) indicate that the decisive influence on the number of contralateral rotations was mainly exerted by L-DOPA, even though after the combined administration of sil2+L-DOPA, their number was higher than after L-DOPA alone (Figure 1B). However, results of the two-way ANOVA calculated for the total number of contralateral rotations in the period of 61–120 min (Figure 1C’) showed that mainly the interaction of sil2 and L-DOPA modulated the number of contralateral rotations in this period. Post hoc comparison of the tested groups in the period of 61–120 min shows that the total number of contralateral rotations in rats receiving a single combined dose of sil2+L-DOPA was significantly higher than in those receiving a single dose of L-DOPA alone (Figure 1C).

#### 2.1.2. Acute Treatment with Sildenafil (6 mg/kg) and L-DOPA (12.5 mg/kg)

Results of the two-way ANOVA for repeated measures calculated for the data set shown in Figure 1D, referring to the higher single dose of sildenafil (6 mg/kg; sil6) and L-DOPA (12.5 mg/kg), administered alone or jointly, revealed a significant effect of time and interactions: time × sil6 treatment, time × L-DOPA treatment, and time × sil6 × L-DOPA treatment (Figure 1D’).

Acute combined administration of sil6+L-DOPA, as in the case of sil2+L-DOPA, significantly increased the number of contralateral rotations measured in 10 min intervals in the whole measurement session lasting 120 min compared to the time-dependent effect of L-DOPA alone (Figure 1D). However, the impact of the higher dose of sildenafil in combination with L-DOPA was much stronger than that of the lower dose of sildenafil with L-DOPA already in the first hour of measurement.

Results of the two-way ANOVA calculated for the total number of contralateral rotations in the period of 0–60 min, referring to data presented as a bar graph in Figure 1E, showed that the interaction of sil6 and L-DOPA predominantly modulated the number of contralateral rotations in this period (Figure 1E’). Post hoc comparison showed that in the period of 0–60 min, the total number of contralateral rotations in the group receiving sli6+L-DOPA was significantly higher than in that receiving only L-DOPA (Figure 1E). In turn, results of the two-way ANOVA performed for the total number of contralateral rotations in the period of 61–120 min, referring to data presented in Figure 1F, revealed that mainly L-DOPA was responsible for the increase in their number (Figure 1F’). However, in the group receiving sil6+L-DOPA, the total number of contralateral rotations was still higher than in the group receiving only L-DOPA (Figure 1F).

#### 2.1.3. Chronic Treatment with Sildenafil (2 mg/kg) and L-DOPA (12.5 mg/kg)

A two-way ANOVA for repeated measures calculated for the data set presented in Figure 2A referring to chronic treatment with sil2 and L-DOPA (12.5 mg/kg), alone or in combination, revealed a significant effect of time and the existence of such interactions as time × sil2 treatment, time × L-DOPA treatment, and time × sil2 × L-DOPA treatment (Figure 2A’). Compared to acute treatment with L-DOPA alone, its chronic administration more distinctly increased the number of contralateral rotations (Figure 2A,D). However, chronic combined administration of sil2+L-DOPA, in contrast to the acute effect, decreased the number of contralateral rotations measured in 10 min intervals during the whole measurement session lasting 120 min compared to the effect of L-DOPA alone (Figure 2A).

More precisely, the results of the two-way ANOVA calculated for the total number of contralateral rotations in the period of 0–60 min (Figure 2B’) revealed that the interaction of sil2 and L-DOPA modulated their number. Post hoc comparison showed that in this period, the number of contralateral rotations in the group treated with sil2+L-DOPA was significantly lower than that in the group treated with L-DOPA alone (Figure 2B). However, a two-way ANOVA performed for the period of 61–120 min (Figure 2C’) showed only a significant effect of L-DOPA on the number of these rotations. In this period, the total number of contralateral rotations in the group chronically receiving the combination of sil2+L-DOPA was comparable to those receiving L-DOPA alone.

#### 2.1.4. Chronic Treatment with Sildenafil (6 mg/kg) and L-DOPA (12.5 mg/kg)

A two-way ANOVA for repeated measures calculated for the data set shown in Figure 2D, referring to the chronic treatment with a higher dose of sildenafil (6 mg/kg) and L-DOPA (12.5 mg/kg), alone or in combination, showed a significant effect of time and an interaction of time × L-DOPA treatment, but no interactions of time × sil6 treatment and time × sil6 × L-DOPA treatment (Figure 2D’). The time-dependent changes in the number of contralateral rotations did not differ between the group treated chronically with L-DOPA alone and that treated with the sil6+L-DOPA combination (Figure 2D).

Consistently, a two-way ANOVA calculated for the total number of contralateral rotations in the periods of 0–60 min and 61–120 min revealed only a significant treatment effect of L-DOPA, a lack of the effect of sil6 treatment, and no interaction between these two drugs (Figure 2E’,F’). The total number of contralateral rotations in both these periods remained similar in the group treated chronically with L-DOPA alone and that treated with the sil6+L-DOPA combination (Figure 2E,F).

### 2.2. The Effect of Chronic Administration of Sildenafil and L-DOPA on DA Metabolism in the Striatum and Substantia Nigra of Unilaterally 6-OHDA-Lesioned Rats

The HPLC method for determining monoamines and their metabolites in different brain structures has been used in our laboratory for several years, and the best chromatographic conditions were selected and developed to perform this analysis. In the present study, chromatographic analysis of the contents of DA, 5-HT, and their metabolites (DOPAC, HVA, 5-HIAA) was performed on two separate sets of rat groups with the unilateral 6-OHDA-induced lesion of the nigrostriatal dopaminergic pathway, each of which represented four groups of eight animals. To maintain the same conditions during chromatographic procedures, the striatal and nigral tissue samples from these two sets were analyzed as follows. In the first set, all ipsi- (32) and contralateral (32) striatal tissue samples from unilaterally 6-OHDA-lesioned rats treated chronically i.p. for two weeks with the vehicle, sil2, L-DOPA 12.5 and the combination of sil2+L-DOPA were analyzed in one session by comparing the peak area of the tested samples with freshly prepared standards, run on the day of analysis. In the next session, all ipsi- (32) and contralateral (32) nigral tissue samples from the same groups were analyzed similarly. In the second set, all ipsi- (32) and contralateral (32) striatal tissue samples as well as all ipsi- (32) and contralateral (32) nigral tissue samples from unilaterally 6-OHDA-lesioned rats, treated chronically i.p. for two weeks with the vehicle, sil6, L-DOPA 12.5 and the combination of sil6+L-DOPA, were analyzed analogously. Figure S1 shows representative chromatograms for DA, 5-HT, and their metabolites in the homogenate of the ipsi- and contralateral striatal tissue sample as well as in the homogenate of the ipsi- and contralateral nigral tissue sample from unilaterally 6-OHDA-lesioned rats treated chronically i.p. with the vehicle, with the indicated the approximated retention times of measured substances that are as follows: for DOPAC = 4.26; DA = 5.31, 5-HIAA = 7.98, HVA = 12.53, and 5-HT = 14.2 min. Symmetrical peaks and the absence of any co-eluting peaks indicate a good specificity of the method.

**Figure 3:**
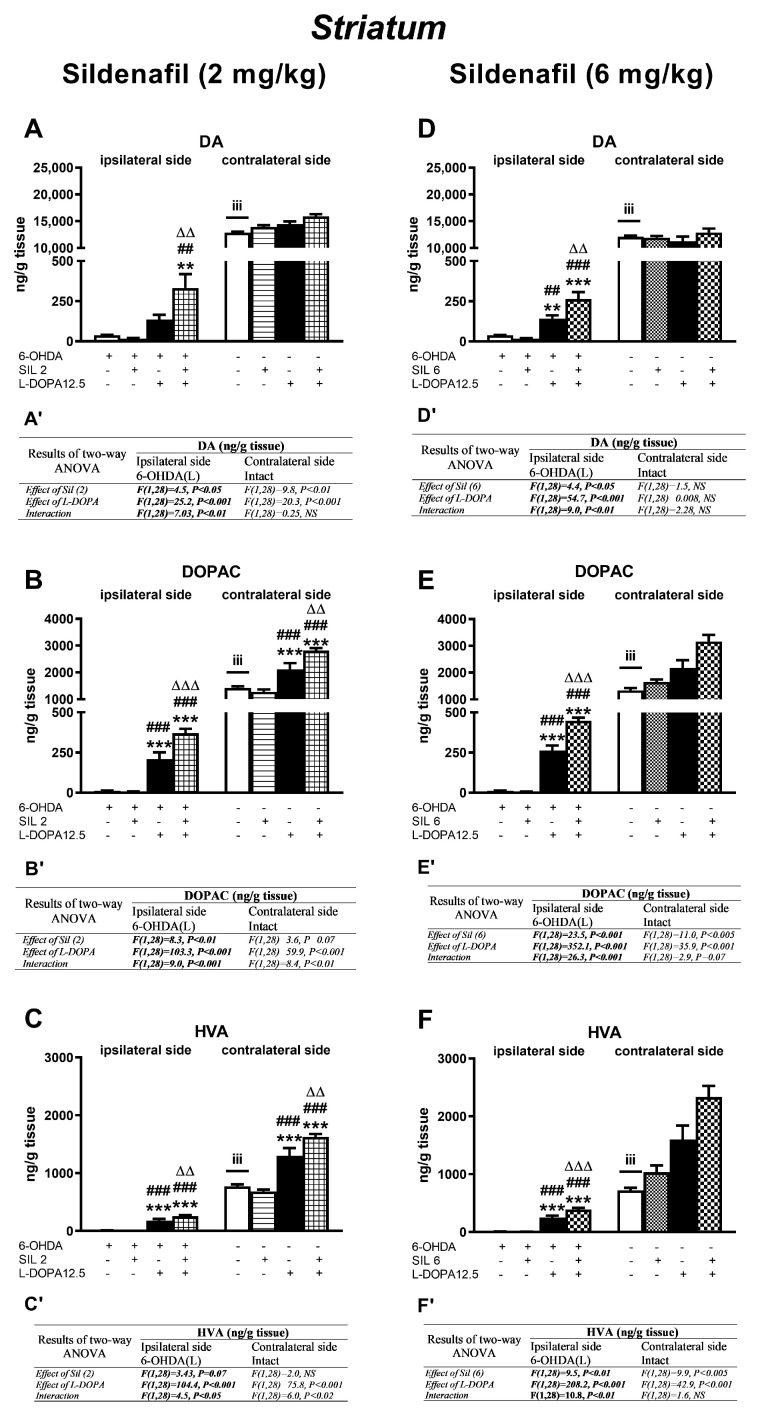
Concentrations of dopamine (DA) and its metabolites (DOPAC, HVA) in the ipsi- and contralateral striatum (STR) of rats with the unilateral lesion to the nigrostriatal dopaminergic pathway induced by 6-OHDA, 1 h after the last chronic doses of sildenafil (2 or 6 mg/kg, i.p.) and L-D-DOPA (12.5 mg/kg, i.p.), administered alone or in combination. The data are presented as the mean ± SEM; the number of rats in each experimental group was n = 8. Statistical analysis of the data presented in (**A**–**F**) was performed using a two-way ANOVA and its effects are included below as (**A’**–**F’**). The abbreviation of NS denotes a nonsignificant effect. Symbols indicate the significance of differences according to the Newman–Keuls post hoc test: ** *p* < 0.01, *** *p* < 0.001 vs. 6-OHDA; ^##^ *p* < 0.01, ^###^ *p* < 0.001 vs. 6-OHDA+sil2- or 6-OHDA+sil6; ^∆∆^ *p* < 0.01, ^∆∆∆^ *p* < 0.001 vs. 6-OHDA+L-DOPA-treated groups, within the corresponding ipsi- or contralateral side. These symbols were marked above the appropriate bars only when there was a significant interaction between the studied drugs. Letters indicate the significance of differences determined by Student’s *t*-test for dependent samples: ^iii^ *p* < 0.001 vs. ipsilateral 6-OHDA-lesioned side.

**Figure 4:**
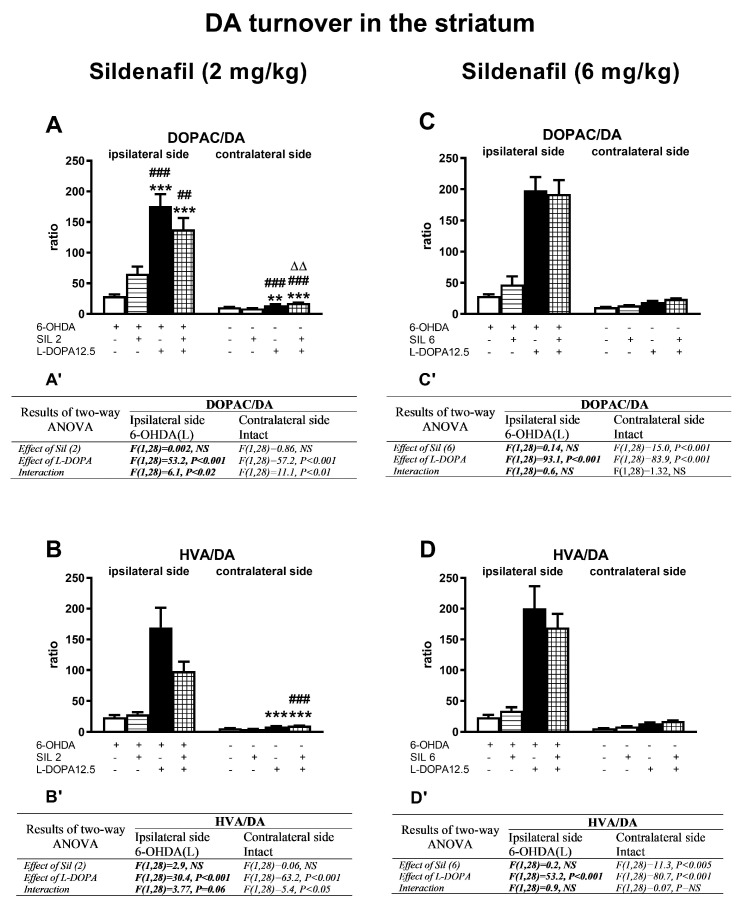
The impact of chronic treatment with sildenafil (2 or 6 mg/kg, i.p.) and L-DOPA (12.5 mg/kg, i.p.), alone or in combination, on the rate of DA catabolism in the ipsi- and contralateral STR of rats with a unilateral lesion of the nigrostriatal dopaminergic pathway induced by 6-OHDA, assessed as DOPAC-to-DA and HVA-to-DA concentration ratios. The data are presented as the mean ± SEM; the number of rats in each experimental group was n = 8. Statistical analysis of the data presented in (**A**–**D**) was performed using a two-way ANOVA and its effects are included below as (**A’**–**D’**). The abbreviation of NS denotes a nonsignificant effect. Symbols indicate the significance of differences according to the Newman–Keuls post hoc test: ** *p* < 0.01, *** *p* < 0.001 vs. 6-OHDA; ^##^ *p* < 0.01, ^###^ *p* < 0.001 vs. 6-OHDA+sil2-; ^∆∆^ *p* < 0.01 vs. 6-OHDA+L-DOPA-treated groups, within the corresponding ipsi- or contralateral side. These symbols were marked above the appropriate bars only when there was a significant interaction between the studied drugs.

**Figure 5:**
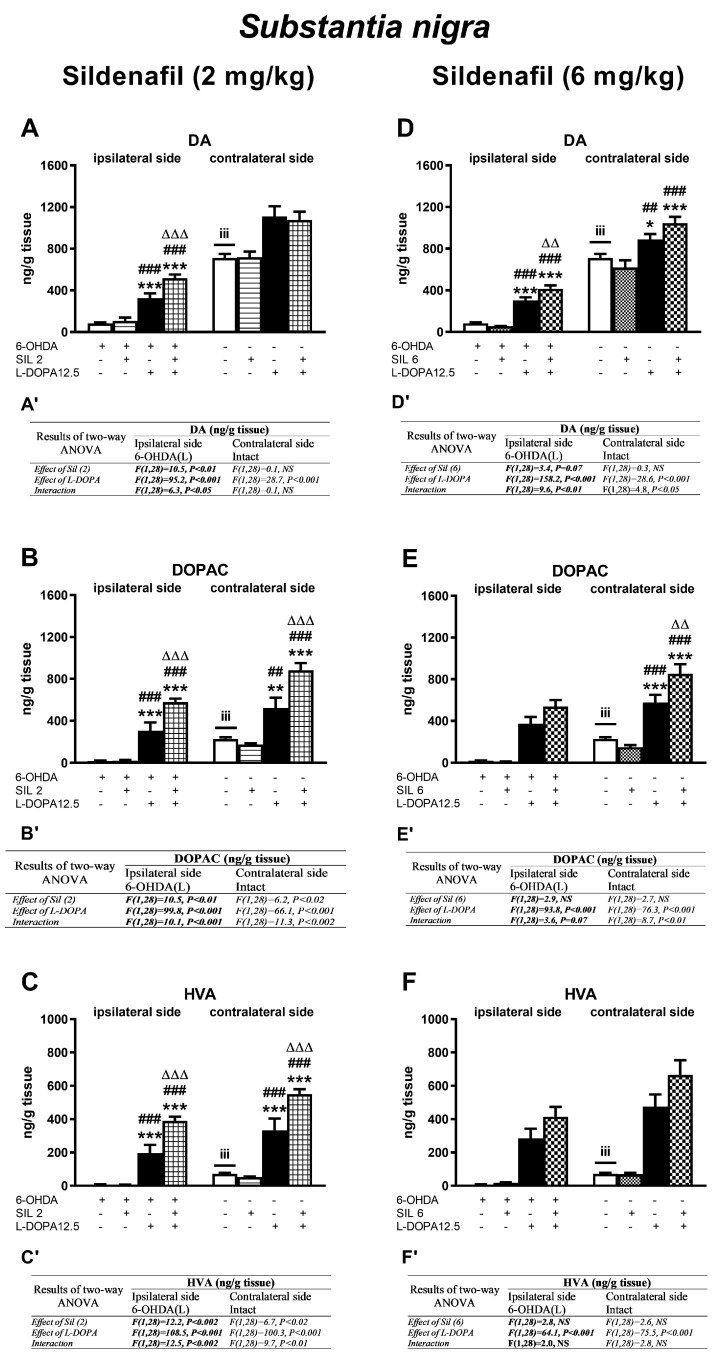
Concentrations of dopamine and its metabolites (DOPAC, HVA) in the ipsi- and contralateral substantia nigra (SN) of rats with the unilateral lesion to the nigrostriatal dopaminergic pathway induced by 6-OHDA, 1 h after the last chronic doses of sildenafil (2 or 6 mg/kg, i.p.) and L-D-DOPA (12.5 mg/kg, i.p.), administered alone or in combination. The data are presented as the mean ± SEM; the number of rats in each experimental group was n = 8. Statistical analysis of the data presented in (**A**–**F**) was performed using a two-way ANOVA and its effects are included below as (**A’**–**F’**). The abbreviation of NS denotes a nonsignificant effect. Statistical analysis of the obtained data was performed using a two-way ANOVA. Symbols indicate the significance of differences according to the Newman–Keuls post hoc test: * *p* < 0.05, ** *p* < 0.01, *** *p* < 0.001 vs. 6-OHDA; ^##^ *p* < 0.01, ^###^ *p* < 0.001 vs. 6-OHDA+sil2- or 6-OHDA+sil6-; ^∆∆^ *p* < 0.01, ^∆∆∆^ *p* < 0.001 vs. 6-OHDA+L-DOPA-treated groups, within the corresponding ipsi- or contralateral side. These symbols were marked above the appropriate bars only when there was a significant interaction between the studied drugs. Letters indicate the significance of differences determined by Student’s *t*-test for dependent samples: ^iii^ *p* < 0.001 vs. ipsilateral 6-OHDA-lesioned side.

**Figure 6:**
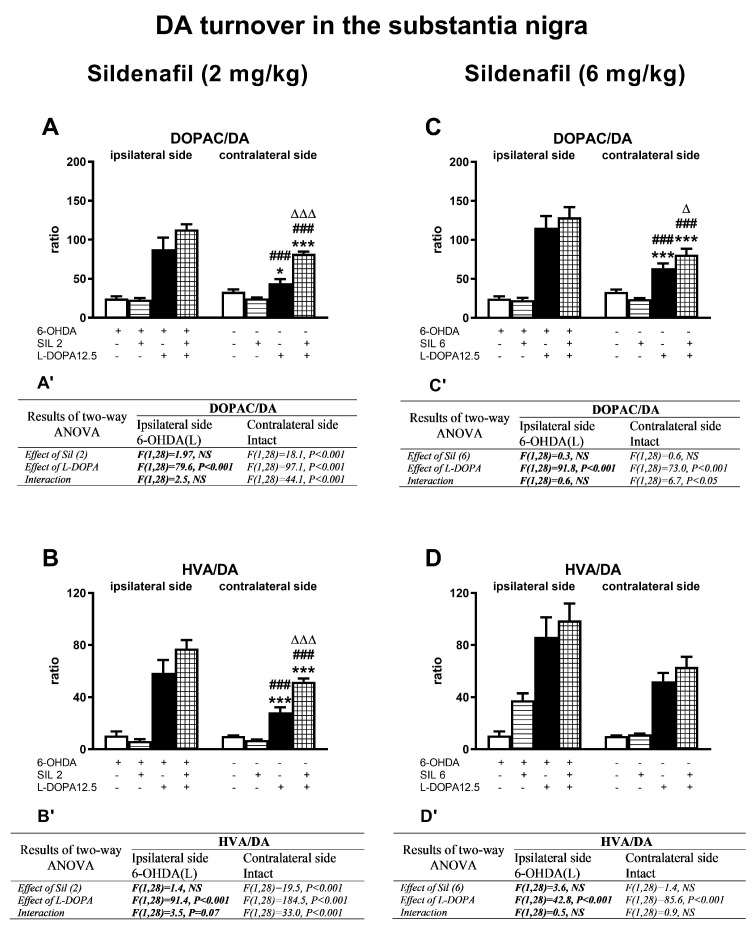
The impact of chronic treatment with sildenafil (2 or 6 mg/kg, i.p.) and L-DOPA (12.5 mg/kg, i.p.), alone or in combination, on the rate of DA catabolism in the ipsi- and contralateral SN of rats with a unilateral lesion of the nigrostriatal dopaminergic pathway induced by 6-OHDA, assessed as the DOPAC-to-DA or HVA-to-DA concentration ratios. The data are presented as the mean ± SEM; the number of rats in each experimental group was n = 8. Statistical analysis of the data presented in (**A**–**D**) was performed using a two-way ANOVA and its effects are included below as (**A’**–**D’**). The abbreviation of NS denotes a nonsignificant effect. Symbols indicate the significance of differences according to the Newman–Keuls post hoc test, * *p* < 0.05, *** *p* < 0.001 vs. 6-OHDA; ^###^ *p* < 0.001 vs. 6-OHDA+sil2- or 6-OHDA+sil6-: ^∆^ *p* < 0.05; ^∆∆∆^ *p* < 0.001 vs. 6-OHDA+L-DOPA-treated groups, within the corresponding ipsi- or contralateral side. These symbols were marked above the appropriate bars only when there was a significant interaction between the studied drugs.

Concentrations of DA and its metabolites (DOPAC, HVA) in the ipsi- and contralateral striatum (STR) of the tested groups, determined 1 h after the last doses of the studied drugs, are shown in Figure 3. Additionally, metabolic indexes such as the DOPAC-to-DA and HVA-to-DA concentration ratios, illustrating the rate of the MAO-dependent oxidative pathway of DA catabolism and the total DA catabolism are shown in Figure 4. In turn, concentrations of DA and its metabolites (DOPAC, HVA) in the ipsi- and contralateral substantia nigra (SN) as well as metabolic indexes illustrating DA catabolism in this brain structure, are shown in Figure 5 and Figure 6. The F values for the two-way ANOVA performed for the concentrations of DA and its metabolites in the STR and SN, and for the metabolic indexes in these brain structures are presented in Figure 3, Figure 4, Figure 5 and Figure 6.

#### 2.2.1. Changes in Dopamine and Its Metabolite Concentrations in the Striatum

Administration of 6-OHDA at a single dose of 8 μg/4 μL unilaterally into the MFB produced a drastic loss of DA and its metabolites in the ipsilateral STR when compared to the contralateral side (Figure 3A–F). Chronic treatment with sil2 or sil6 alone did not affect the tissue DA concentration in the ipsilateral STR in contrast to 12.5 mg/kg of L-DOPA alone, which increased the tissue DA content compared to the DA concentration in the ipsilateral STR of 6-OHDA-lesioned rats receiving the vehicle, sil2, or sil6 (Figure 3A,D).

Moreover, chronic combined treatment with sil2+L-DOPA or sil6+L-DOPA produced markedly higher increases in the tissue DA contents in the ipsilateral STR than in the group of rats receiving L-DOPA alone (Figure 3A,D). Furthermore, a significant interaction was found between sil2 and L-DOPA (12.5) or Sil6 and L-DOPA (12.5) regarding DA content, indicating that the sildenafil affected L-DOPA-derived DA level in the ipsilateral STR (Figure 3A’,D’). However, no such interaction was found for the DA concentration after the administration of sil2+L-DOPA in the contralateral STR, although both sil2 and L-DOPA affected its level (Figure 3A’,D’).

Parallel to the enhancement of the tissue DA content, the administration of L-DOPA alone or the combinations of sil2+L-DOPA and sil6+L-DOPA also increased concentrations of the intraneuronal DA metabolite DOPAC and the total DA metabolite HVA compared to the 6-OHDA-lesioned rats receiving the vehicle, sil2 or sil6 alone, both in the ipsi- and contralateral STR, respectively (Figure 3B,C). Significant interactions between Sil2 and L-DOPA regarding concentrations of these DA metabolites on both sides of the STR were also found (Figure 3B’,C’). In fact, after the combined administration of sil2+L-DOPA, the levels of DOPAC and HVA on both sides of the STR were significantly higher than in the group receiving L-DOPA alone (Figure 3B,C).

In turn, after the combined administration of sil6+L-DOPA, the levels of DOPAC and HVA were significantly higher than in the group receiving L-DOPA alone only in the ipsilateral STR (Figure 3E,F) in which a significant interaction between these drugs was also observed (Figure 3E’,F’). However, in the contralateral STR, although both L-DOPA alone and the combined administration of sil6+L-DOPA significantly increased the levels of DOPAC and HVA (Figure 3E,F) there was no interaction between these drugs for the levels of these DA metabolites (Figure 3E’,F’).

Regarding DA turnover in the ipsi- and contralateral STR, treatment with L-DOPA alone or with sil2+L-DOPA and sil6+L-DOPA combinations accelerated DA catabolism. In these groups of rats, the values of metabolic indexes expressed as DOPAC/DA and HVA/DA were generally significantly higher than the in 6-OHDA-lesioned rats receiving the vehicle, sil2, or sil6 alone (Figure 4A–D).

However, in the ipsilateral STR of rats receiving sil2+L-DOPA, the values of the metabolic indexes of DOPAC/DA and HVA/DA were significantly lower than in the group treated with L-DOPA alone, but in contrast, in the contralateral STR of rats receiving sil2+L-DOPA, the value of the metabolic index DOPAC/DA was markedly higher than in the group receiving L-DOPA alone, while the values of the HVA/DA indexes were similar in both groups (Figure 4A,B). Furthermore, there were significant interactions between sil2 and L-DOPA for DOPAC/DA and HVA/DA metabolic indexes in the contralateral STR (Figure 2A’,B’). These results indicate that the catabolism of L-DOPA-derived DA in the presence of Sil2 is differentially modulated in the ipsi- and contralateral STR, which may have some implications for asymmetric behavior.

As for the metabolic indexes of DOPAC/DA and HVA/DA, in the groups receiving L-DOPA alone or the combination of sil6+L-DOPA, their values did not differ between these groups both in the ipsi- and contralateral STR (Figure 4C,D). In the ipsilateral STR, only L-DOPA affected the values of these indexes (Figure 4C’,D’). Interestingly, in the contralateral STR, both sil6 and L-DOPA administered alone affected these indexes, but there were no interactions between these drugs (Figure 4C’,D’). The latter results suggest that in the contralateral STR, the catabolism of L-DOPA-derived DA is independently regulated by Sil6 and L-DOPA.

#### 2.2.2. Changes in Dopamine and Its Metabolite Concentrations in the Substantia Nigra

Analogous types of changes in the concentrations of DA and its metabolites as those seen in the STR, after the administration of sil2 and L-DOPA, alone or in combination, were also found in the ipsi- and contralateral SN (Figure 5A–C), except for the DA content in the contralateral SN (Figure 5A). The concentrations of DA in the groups of rats receiving L-DOPA alone or the Sil2+L-DOPA combination in the contralateral SN significantly increased compared to their contents in the corresponding groups of rats receiving the vehicle or sil2 alone (Figure 5A). As shown by a two-way ANOVA, only L-DOPA was responsible for these increases (Figure 5A’).

Regarding the impact of sil6 and L-DOPA on the DA concentration in the SN, both the administration of L-DOPA alone or the sil6+L-DOPA combination significantly increased the DA content in the ipsi- and contralateral SN (Figure 5D). As shown by the two-way ANOVA, the interaction between Sil6 and L-DOPA played a significant role in the increase in DA concentration in the ipsi- and contralateral SN in the group of rats receiving these two drugs jointly (Figure 5D’). Furthermore, in the ipsi- and contralateral SN, similar to those in the STR, the levels of DA metabolites, DOPAC and HVA, in the groups of rats receiving L-DOPA alone or the sil6+L-DOPA combination were markedly increased compared to their contents in the corresponding groups of 6-OHDA-lesioned rats receiving the vehicle or sil(6) alone (Figure 5E,F). As shown by a two-way ANOVA, only L-DOPA was responsible for the increase in the DOPAC content in the ipsilateral SN and HVA in the ipsi- and contralateral SN (Figure 5E’,F’) as opposed to the DOPAC content in the contralateral SN, which was increased both by L-DOPA itself and as a result of the interaction of sil6 and L-DOPA (Figure 5E’).

Similarly to the striatum, DA catabolism in the ipsi- and contralateral SN, assessed as the DOPAC/DA and HVA/DA metabolic indexes, was intensified after the administration of L-DOPA alone and the combination of SIL2+L-DOPA or SIL6+L-DOPA (Figure 6A–D). As shown by a two-way ANOVA, only L-DOPA contributed to the increases in DOPAC/DA and HVA/DA metabolic indexes in the ipsilateral SN (Figure 6A’–D’). Nevertheless, the mean values of these indexes in the ipsilateral SN did not differ significantly between groups receiving L-DOPA alone or the combinations of sil2+L-DOPA or sil6+L-DOPA.

On the other hand, in the contralateral SN of rats receiving sil2+L-DOPA, the values of the metabolic indexes of DOPAC/DA and HVA/DA were significantly higher than in the group treated with L-DOPA alone (Figure 6A,B). Furthermore, in the contralateral SN, there were significant interactions between sil2 and L-DOPA for the DOPAC/DA and HVA/DA metabolic indexes (Figure 6A’,B’).

As for the effects of the treatment with Sil6 and L-DOPA in the contralateral SN, only the value of the metabolic index DOPAC/DA in the group receiving sil6+L-DOPA was significantly higher than in the group receiving L-DOPA alone (Figure 6C), but the values of the HVA/DA indexes were at a similar level in both these groups (Figure 6D,D’).

#### 2.2.3. Changes in Serotonin and Its Metabolite Concentrations in the Striatum

**Figure 7:**
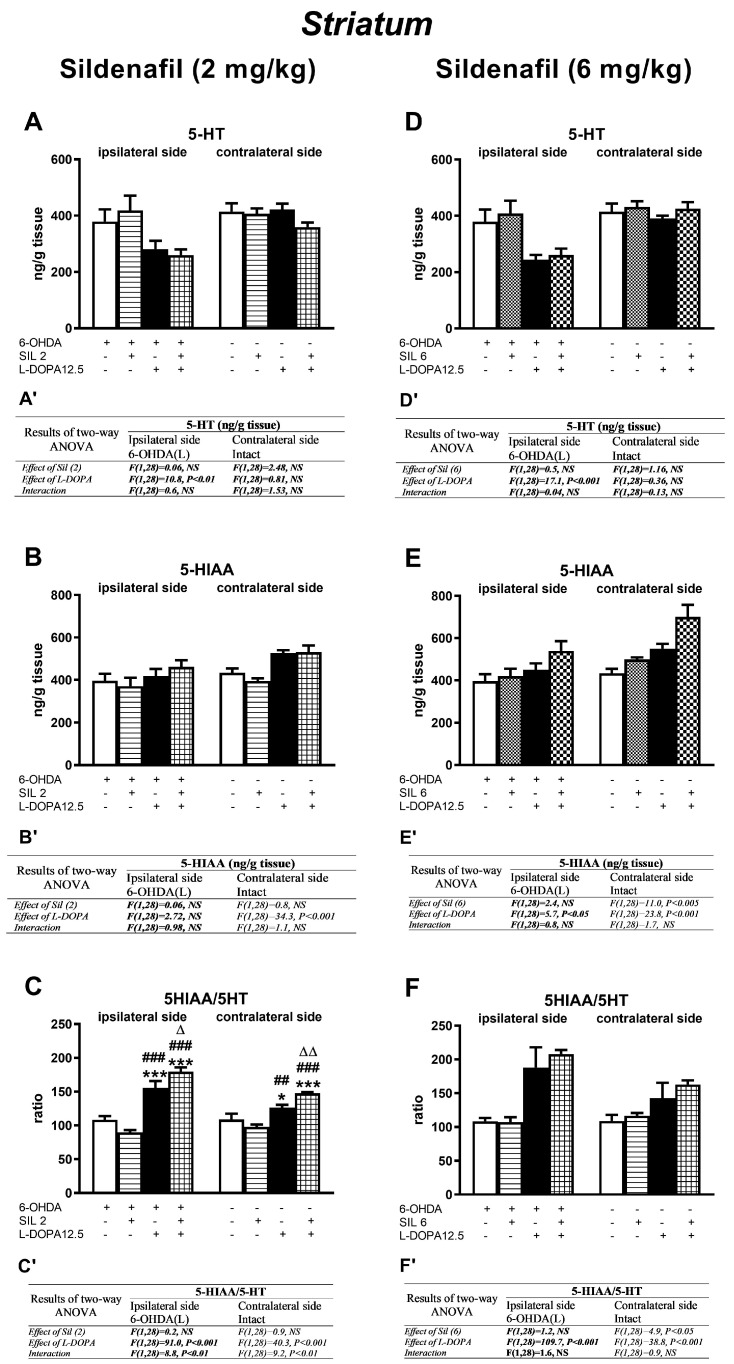
The impact of chronic treatment with sildenafil (2 or 6 mg/kg, i.p.) and L-DOPA (12.5 mg/kg, i.p.), alone or in combination, on concentrations of serotonin (5-HT) and its metabolite (HIAA), and on the rate of 5-HT catabolism assessed as the HIAA-to-5-HT concentration ratio, in the ipsi- and contralateral STR of rats with the unilateral lesion to the nigrostriatal dopaminergic pathway induced by 6-OHDA. The data are presented as the mean ± SEM, and the number of rats in each experimental group was n = 8. Statistical analysis of the data presented in (**A**–**F**) was performed using a two-way ANOVA and its effects are included below as (**A’**–**F’**). The abbreviation of NS denotes a nonsignificant effect. Symbols indicate the significance of differences according to the Newman–Keuls post hoc test: * *p* < 0.05, *** *p* < 0.001 vs. 6-OHDA; ^##^ *p* < 0.01, ^###^ *p* < 0.001 vs. 6-OHDA+sil2-; ^∆^ *p* < 0.05, ^∆∆^ *p* < 0.01 vs. 6-OHDA+L-DOPA-treated groups, within the corresponding ipsi- or contralateral side. These symbols were marked above the appropriate bars only when there was a significant interaction between the studied drugs.

As shown by a two-way ANOVA, only L-DOPA affected the 5-HT content in the ipsilateral STR by reducing its level in the groups of rats treated with L-DOPA alone and with the combinations of sil2+L-DOPA and sil6+L-DOPA, as opposed to the contralateral STR, in which no such effect of L-DOPA was observed (Figure 7A,D).

Furthermore, in the ipsilateral STR of rats treated with sil2 and L-DOPA, alone or in combination, no changes in the concentration of the 5-HT metabolite HIAA were observed in any of the tested groups (Figure 7B). Nevertheless, in the contralateral STR, L-DOPA affected the concentration of 5-HIAA, which was increased in the groups of rats receiving L-DOPA alone or the sil2+L-DOPA combination (Figure 7B,B’).

However, in the groups of rats treated with sil6+L-DOPA, the 5-HIAA concentration in the ipsilateral STR was influenced only by L-DOPA, while in the contralateral STR, it was influenced by both L-DOPA and Sil6, but there was no interaction between these drugs (Figure 7E,E’). Finally, the concentration of 5-HIAA in the ipsi- and contralateral STR was particularly increased in the group of rats receiving the sil6+L-DOPA combination (Figure 7E).

The rate of 5-HT catabolism, expressed as the 5-HIAA/5-HT metabolic index, was increased in all groups treated with L-DOPA alone or its combination with Sil2 or Sil6 (Figure 7C,F). In the ipsi- and contralateral STR of rats receiving sil2+L-DOPA, the 5-HIAA/5-HT metabolic index was significantly higher than in the group receiving L-DOPA alone (Figure 7C), and this effect was due to the interaction of Sil2 and L-DOPA (Figure 7C’). However, in the ipsi- and contralateral STR of rats receiving sil6+L-DOPA, the 5-HIAA/5-HT metabolic index was as high as in the group receiving L-DOPA alone (Figure 7F), and there was no interaction of Sil6 and L-DOPA (Figure 7F’).

#### 2.2.4. Changes in Serotonin and Its Metabolite Concentrations in the Substantia Nigra

**Figure 8:**
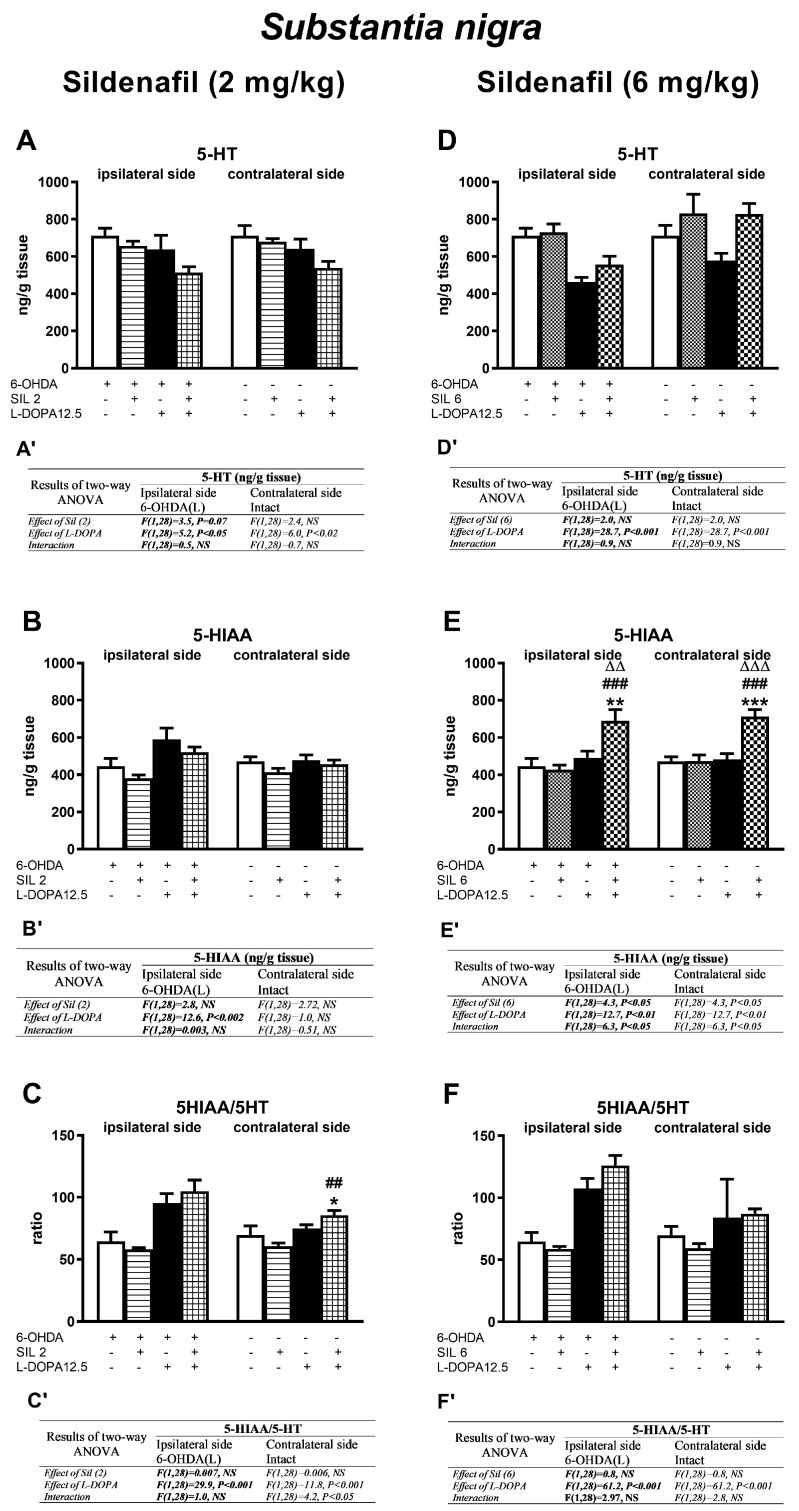
The impact of chronic treatment with sildenafil (2 or 6 mg/kg, i.p.) and L-DOPA (12.5 mg/kg, i.p.), alone or in combination, on concentrations of serotonin (5-HT) and its metabolite (HIAA), and on the rate of 5-HT catabolism assessed as the HIAA-to-5-HT concentration ratio, in the ipsi- and contralateral SN of rats with the unilateral lesion to the nigrostriatal dopaminergic pathway induced by 6-OHDA. The data are presented as the mean ± SEM; the number of rats in each experimental group was n = 8. Statistical analysis of the data presented in (**A**–**F**) was performed using a two-way ANOVA and its effects are included below as (**A’**–**F’**). The abbreviation of NS denotes a nonsignificant effect. Symbols indicate the significance of differences according to the Newman–Keuls post hoc test: * *p* < 0.05, ** *p* < 0.01, *** *p* < 0.001 vs. 6-OHDA; ^##^ *p* < 0.01, ^###^ *p* < 0.001 vs. 6-OHDA+sil6-; ^∆∆^ *p* < 0.01, ^∆∆∆^ *p* < 0.001 vs. 6-OHDA+L-DOPA-treated groups, within the corresponding ipsi- or contralateral side. These symbols were marked above the appropriate bars only when there was a significant interaction between the studied drugs.

As shown by a two-way ANOVA, only L-DOPA influenced the 5-HT content in the ipsi- and contralateral SN (Figure 8A,A’,D,D’), reducing its level in the sil2+L-DOPA treated group on both sides (Figure 8A), and in the group treated with sil6+L-DOPA, only in the ipsilateral SN (Figure 8D).

As for the concentration of the 5-HT metabolite 5-HIAA, L-DOPA increased its level only in the ipsilateral SN of rats treated with L-DOPA alone or with the sil2+L-DOPA combination (Figure 8B,B’). In turn, in the groups of rats treated with Sil6 and L-DOPA, alone or in combination, this effect was mediated by both Sil6 and L-DOPA (Figure 8E,E’). Moreover, a significant interaction was found between Sil6 and L-DOPA, and the most pronounced increase in the 5-HIAA concentration was observed in the group receiving these drugs jointly (Figure 8E).

The rate of 5-HT catabolism in the SN was increased in the group of rats receiving L-DOPA alone or the combination of sil2+L-DOPA or sil6+L-DOPA on both sides of this structure (Figure 8C,C’,F,F’). The intensification of 5-HT catabolism was mainly due to the effect of L-DOPA, and only in the contralateral SN of the group of rats receiving the Sil2+L-DOPA combination, it was the result of the interaction of these two drugs (Figure 8C,C’).

## 3. Discussion

The results of the present study show that the effects of the combined administration of sil2+L-DOPA or sil6+L-DOPA were markedly different compared to the administration of L-DOPA alone, both after acute and chronic treatment. After a single administration of sil2+L-DOPA, a more conspicuous increase in the number of contralateral rotations was observed than after L-DOPA alone. However, this effect was biphasic and in the first hour of measurement the increase was rather small, but in the second hour it was significantly greater. In contrast, acute administration of sil6+L-DOPA caused an immediate and much stronger increase in the number of contralateral rotations compared to the action of L-DOPA alone, already visible in the first hour of measurement. In the second hour of measurement, the increase in the number of contralateral rotations continued, although it was slightly weaker. The comparison of the effects of Sil2+L-DOPA with sil6+L-DOPA over time clearly shows that the action of a higher dose of sildenafil on the L-DOPA-induced rotation behavior was much stronger and immediate, while the action of a lower dose was weaker and delayed. These results suggest that significantly greater increases in the number of contralateral rotations after a single combined administration of sildenafil and L-DOPA than after L-DOPA alone may be related to the rise in the concentration of cGMP due to the sildenafil-mediated inhibition of PDE5 enzymatic activity. Moreover, as the unilateral 6-OHDA-induced lesion of DA neurons in the SN caused an increase in cAMP [32,33], but a decrease in cGMP concentrations in the ipsilateral STR [30,33], adding a single dose of sildenafil to the first dose of L-DOPA thereby increasing cGMP in the STR, especially on its ipsilateral side, may increase the number of contralateral rotations. Furthermore, these behavioral data indicate that when L-DOPA treatment is initiated, the addition of a low to moderate dose of sildenafil to the standard therapy with L-DOPA alone may enhance the effectiveness of L-DOPA in improving motor functions.

However, as our study shows, after long-term combined administration of sildenafil and L-DOPA, the presented picture of the asymmetric behavior of rats seen after a single administration of these drugs has changed significantly. Interestingly, chronic combined administration of sil2+L-DOPA significantly reduced the number of contralateral rotations, especially during the first hour of measurement, compared to their number after long-term treatment with L-DOPA alone. This effect was not observed under the chronic combined administration of sil6+L-DOPA compared to the chronic administration of L-DOPA alone; in both these groups, the number of contralateral rotations analyzed over a 2 h session was comparable.

The results of the present study can be compared to the results of our previous work [48], which investigated the effect of the nitric oxide donor molsidomine (2 mg/kg), administered alone or in combination with L-DOPA (25 or 12.5 mg/kg) on rotational behavior. In that study, a NO donor was used to compensate for the deficiency in the activity of nNOS and to stimulate the modulation of the NO-sGC-cGMP signaling pathway. The results presented in that study showed that chronic co-administration of 2 mg/kg of molsidomine with 25 or 12.5 mg/kg of L-DOPA reduced the number of contralateral rotations compared to the corresponding doses of L-DOPA alone. The convergence of behavioral effects regulating rotational behavior induced by the long-term combined administration of either 2 mg/kg of molsidomine with L-DOPA (12.5 mg/kg) or 2 mg/kg of sildenafil with the same dose of L-DOPA indicates the existence of a common mechanism that was responsible for this effect. It seems that cGMP is one of the factors involved in this mechanism, thus its concentration should increase both after the chronic administration of the NO donor molsidomine and the PDE5 inhibitor sildenafil.

On the other hand, it is worth reminding that the chronic administration of L-DOPA, the most effective symptomatic treatment of motor deficits in PD patients and animal models, gradually increases rotational behavior towards the side contralateral to the lesion and the intensity of this process is directly dependent on the hypersensitivity of the striatal postsynaptic DA receptors, which results from DA depletion on the lesioned side, and the used dose of L-DOPA [9,49,50]. Moreover, as mentioned in the Introduction section, in non-dyskinetic rats, chronic L-DOPA treatment drastically reduced the cGMP concentration only in the contralateral STR, but had no effect on its level in the ipsilateral STR, while, in parallel, increasing the cAMP content in both the ipsi- and contralateral STR [33,34]. Therefore, it seems reasonable to explain the observed chronic behavioral effects after adding a low dose of sildenafil or molsidomine to standard L-DOPA therapy in the context of an increase in cGMP concentration, which, by balancing the deficit of this nucleotide in the contralateral STR, may lead to a reduction in the number of contralateral rotations.

For a long time, the ability of L-DOPA to induce contralateral rotations in unilaterally 6-OHDA-lesioned rats was treated as a valuable marker reflecting its antiparkinsonian activity [49,50]. However, currently, contralateral rotations are considered rather a marker of locomotor activity [51]. In this context, the reduction in the number of contralateral rotations after the combined chronic treatment with sil2+L-DOPA(12.5) or molsidomine(2)+L-DOPA(12.5) could be interpreted, on the one hand, as a weakening of the antiparkinsonian effect of L-DOPA, and on the other hand, as a weakening of excessive locomotor activity at the peak of L-DOPA action. The second explanation seems to be more reliable. A third potential explanation for the lower number of contralateral rotations after combined treatment with the above-mentioned combinations of the tested drugs compared to the effect of L-DOPA alone is the reduction in excessive asymmetry in the action of L-DOPA. Summing up of the behavioral part of this study, from a clinical point of view, the results obtained after the long-term use of the tested drugs are more important for the therapy than those associated with their acute administration.

Due to the above conclusion, in the biochemical part of this study, the determination of tissue concentrations of DA and 5-HT, and their metabolites, in the STR and SN was performed only in groups of rats chronically treated with sildenafil at doses of 2 mg/kg or 6 mg/kg, and L-DOPA at a dose of 12.5 mg/kg, alone or in combination, 1 h after their last doses. The obtained results showed that the tissue concentrations of L-DOPA-derived DA in the ipsilateral STR and SN were significantly increased in the groups of rats treated with L-DOPA alone and in those treated with sil2+L-DOPA or sil6+L-DOPA combinations. Moreover, the increase in DA contents was considerably higher in the group receiving a combination of sil2+L-DOPA or sil6+L-DOPA than in the group treated with L-DOPA alone. However, these increases in the tissue DA concentrations both in the ipsilateral STR and in the ipsilateral SN were not reflected in rotational behavior, especially in the group of rats receiving the sil2+L-DOPA combination, in which the number of contralateral rotations was significantly lower than in the group receiving L-DOPA alone.

It is worth noting, however, that an increase in the tissue concentration of DA does not necessarily translate directly into the extracellular, functional pool of this neurotransmitter. In general, the extracellular concentration of DA depends on both the rate of its release into the extracellular space and the efficiency of its reuptake from this space, as well as on the rate of DA catabolism. However, all these processes are impaired by the unilateral 6-OHDA-induced lesion of DA neurons in the SN and chronic L-DOPA treatment. In the ipsilateral lesioned STR and SN, the conversion of exogenous L-DOPA to DA, as well as its storage in the synaptic vesicles and its release to the synaptic cleft, occur predominantly in the serotonergic terminals [52,53,54,55,56] which densely innervate the SN and moderately innervate the STR [57,58,59,60]. However, in the intact contralateral STR and SN, the conversion of L-DOPA endogenously synthesized from L-tyrosine and that of exogenous origin occurs in the striatal DA terminals and the cell bodies and dendrites of DA neurons in the SN. Moreover, the appearance of contralateral rotations in rats with a unilateral 6+OHDA lesion after L-DOPA administration is associated with the increased DA release in both the contralateral STR and the contralateral SN [61]. Therefore, in our study, to assess the impact of DA catabolism on the level of the extracellular, functional DA pool, we analyzed the indexes characterizing DA catabolism in the STR and SN of rats treated with L-DOPA alone or the combinations of sil2+L-DOPA and sil6+L-DOPA, both on the ipsilateral and contralateral sides. This analysis may be crucial for the interpretation of the difference in rotational behavior after the administration of the sil2+L-DOPA combination compared to the effect of L-DOPA alone, and the lack of this difference after the administration of the sil6+L-DOPA combination compared with the effect of L-DOPA alone.

L-DOPA administered chronically, alone or in combination with sildenafil, increased DA catabolism, as assessed based on the DOPAC/DA index reflecting intracellular oxidative, MAO-dependent DA catabolism and on the HVA/DA index reflecting the total DA catabolism involving both the intracellular and extracellular pool of DA. In the ipsilateral STR, the attenuation of DA catabolism, as assessed by a decrease in the catabolic DOPAC/DA index and a strong downward trend in the HVA/DA index in the group of rats treated with the sil2+L-DOPA combination compared to these indexes in the group treated with L-DOPA alone, may mean that after the combined administration of sil2+L-DOPA, the concentration of the extracellular functional pool of DA could be higher than after the administration of L-DOPA alone. Conversely, in the contralateral STR and SN, the acceleration of DA catabolism, as assessed by an increase in both the catabolic DOPAC/DA and HVA/DA indexes in the group of rats treated with the sil2+L-DOPA combination compared to these indexes in the group treated with L-DOPA alone, may indicate that after the combined administration of sil2+L-DOPA, the concentration of the extracellular functional pool of DA could be lower than after the administration of L-DOPA alone. This potential increase in the concentration of the functional DA pool in the ipsilateral STR with a simultaneous decrease in the concentration of this DA pool in the STR and SN on the contralateral side may explain the reduction in the number of contralateral rotations in the group of rats receiving the sil2+L-DOPA combination compared to the group receiving L-DOPA alone. On the other hand, the lack of difference in rotational behavior in rats treated chronically with the sil6+L-DOPA combination compared to L-DOPA alone could be explained by a similar intensity of DA catabolism in both groups of rats which resulted in comparable DA concentrations in the ipsilateral and contralateral STR and SN.

In light of the above analysis, the reduction in the number of contralateral rotations after the combined administration of a low dose of the PDE5 inhibitor, sildenafil, and L-DOPA could be interpreted as partial compensation for the disturbed functional balance induced by L-DOPA alone. The partial compensation of this imbalance may be of particular importance in the SN, which, like the STR, is also responsible for the occurrence of contralateral rotations after the administration of L-DOPA [61], and whose reticular part is thought to be particularly involved in the control of balance during gait [62]. Thus, this partial compensation could play some role in improving the postural instability observed in PD patients.

It is also worth mentioning that the catabolism of 5-HT to 5-HIAA in the serotonergic terminals in the STR and SN and the 5-HT release from these terminals may affect the concentration of the extracellular functional DA pool in the examined brain structures. In the serotonergic terminals of the ipsilateral STR and ipsilateral SN, DA-derived from L-DOPA competes with 5-HT for the vesicular monoamine transporter VMAT2 to enter exocytotic vesicles [63,64], which ultimately leads to DA accumulation in 5-HT vesicles, probably at the expense of endogenous 5-HT, which was extensively catabolized by aromatic L-amino acid decarboxylase (AADC) present in the cytoplasm. However, in the contralateral unlesioned STR and SN, L-DOPA is not metabolized in serotonergic terminals and does not interfere with the catabolism and storage of 5-HT in synaptic vesicles. In our study, 5-HT catabolism, as assessed by the 5-HIAA/5-HT index in the ipsi- and contralateral STR of rats treated with the combination of sil2+L-DOPA, was significantly higher than in the group treated with L-DOPA alone. This acceleration in 5-HT catabolism in the ipsilateral striatum of rats treated with the combination of sil2+L-DOPA or L-DOPA alone resulted in a decrease in the tissue concentration of 5-HT and probably also its release, while in the contralateral STR, only in the group of rats receiving sil2+L-DOPA did the increase in 5-HT catabolism lead to a slight downward trend in the 5-HT content. A similar course of 5-HT catabolism was observed in the SN.

In conclusion, the presented behavioral and biochemical studies indicate a potential new mechanism modulating L-DOPA-induced asymmetric behavior in 6-OHDA-lesioned rats in the presence of a PDE5 inhibitor.

### Limitation of the Study

The present research examined the potential interaction of the PD5 inhibitor sildenafil used at a low and moderate dose of 2 and 6 mg/kg in combination with a moderate dose of L-DOPA (12.5 mg/kg) on the rotation behavior as well as on the tissue concentrations of DA, 5-HT and their metabolites (DOPAC, HHA, and 5-HIIA) in the STR and SN of the unilaterally 6-OHDA-induced model of PD. Although most of the presented behavioral and biochemical parameters show the existence of an interaction between the used doses of sildenafil and L-DOPA, the selection of these doses was not confirmed by typical methodologies, such as isobologram or synergistic interaction surfaces, commonly used in pharmacological studies to assess drug–drug interactions, which is undoubtedly a limitation of this study. It is worth noting that the effects of sildenafil on motor functions and monoamine metabolism in the rodent models of PD have not been studied so far. Hence, the selection of sildenafil doses used in the current study was based on experimental data obtained from studies performed in rat and mouse models of Alzheimer’s disease, in which 2 and 6 mg/kg of sildenafil improved cognitive functions [65,66,67].

## 4. Materials and Methods

### 4.1. Drugs

Apomorphine hydrochloride (Cat. No A43930), benserazide hydrochloride (Cat. No B7283), desipramine hydrochloride (DES; Cat. No D3900), 3,4-dihydroxy-L-phenylalanine (L-DOPA, Cat. No D9628), 6-hydroxydopamine hydrochloride (6-OHDA; Cat. No H4381) and L-ascorbic acid (Cat. No A0278) were provided by the Sigma-Aldrich Chemical Company (Steinheim, Germany), sildenafil citrate was gifted by Pfizer Inc. (New York, NY, USA), while ketamine and diazepam were given by Biowet and Polfa (Warszawa, Poland), respectively. If not stated otherwise, all other compounds were provided by the Sigma-Aldrich Chemical Company (Steinheim, Germany).

### 4.2. Animals, Surgery, and Drug Treatment

Experiments were performed on 3-month-old male Wistar rats (Charles River, Sulzfeld, Germany) with initial body weights ranging between 290 and 320 g. They were housed in groups of 5 in large plastic cages, with unlimited access to standard laboratory feed and tap water. The rats were maintained in housing rooms at a temperature of 22 ± 2 °C, a humidity of 45–50%, and under an artificial 12/12 h light/dark cycle. All research tasks have been completed according to the Act on the Protection of Animals Used for Scientific or Educational Purposes of 21 January 2005, reapproved on 15 January 2015 (published in Journal of Laws no 23/2015 item 266, Poland), and in compliance with the Directive of the European Parliament and of the Council of Europe 2010/63/EU of 22 September 2010 on the protection of laboratory animals. The Ethics Committee of the Maj Institute of Pharmacology, Polish Academy of Sciences, in Kraków, Poland, approved the methods used to perform the planned experiments and their course (permit no. 846/2011 of 19 May 2011). During the experiments, efforts were made to limit the suffering of animals and their numbers and to ensure that the results obtained were statistically reliable (3R policy).

To create an animal model of PD, Wistar rats were subjected to stereotaxic surgery during which the selective dopaminergic neurotoxin 6-hydroxydopamine (6-OHDA) was introduced into their brains. Thirty minutes before surgery, rats were injected with desipramine hydrochloride (15 mg/kg i.p.) to inhibit the 6-OHDA-induced degeneration of noradrenergic pathways [68]. Then, rats were lightly anesthetized with a mixture (1:1 *v*/*v*) of ketamine (50 mg/kg) with diazepam (2.5 mg/kg) administered in a volume of 1ml/kg b.w. and were placed in a stereotaxic apparatus. In the anesthetized rats, a stainless steel needle (0.28 mm outer diameter) was inserted unilaterally through a small hole in the skull, the tip of which was placed in the left medial forebrain bundle (MFB). The stereotaxic coordinates according to the atlas of Paxinos and Watson [69] were as follows: A/P = −2.8 mm, L= +1.8 mm, and DV= −8.6 mm. The solution of 6-OHDAwas freshly prepared before surgery. 6-OHDA hydrochloride at a single dose of 8 µg (calculated as the free base) in a volume of 4 µL of sterile redistilled water supplemented with 0.05% of ascorbic acid was slowly injected into the left MFB at a flow rate of 0.5 µL/min using a Hamilton syringe. Control rats received an equivalent volume of the vehicle instead of 6-OHDA. After stopping the infusion, the cannula tip was left in the target site for at least 10 min to allow the diffusion of the toxin or vehicle and then it was slowly retracted. In the first days after surgery, the rats’ recovery process was monitored by checking their weight and observing their general health and behavior. Typically, rats need about 7 days to fully recover, after which they can be used for behavioral studies [70].

On the 13th day after the surgery, rats were tested for rotational behavior in response to administering a low dose of apomorphine (APO; 0.25 mg/kg s.c). Only rats exhibiting more than 100 contralateral turns within 1 h, which, as previously documented [48,71] are characterized by the extensive loss of nigrostriatal neurons, were taken for further behavioral and biochemical studies. On the following day after the APO test, L-DOPA (12.5 mg/kg suspended in propylene glycol, i.p.) and two doses of sildenafil (2 or 6 mg/kg dissolved in saline, i.p.) were administered once daily, alone or in combination, for 14 consecutive days. Benserazide hydrochloride (6.25 mg/kg dissolved in sterile redistilled water i.p.) was injected 30 min before L-DOPA administration, while sildenafil was given 10 min before L-DOPA. Throughout the text, the administration of benserazide + L-DOPA was referred to as the L-DOPA treatment.

### 4.3. Asymmetric Behavior

To quantify rotational behavior [72], each tested rat was placed in a hemispherical Plexiglas bowl (diameter 50 cm) containing sawdust on the bottom. It was fitted with a collar at the height of the forelimbs, connected to an automated rotameter system, [73] equipped with a computer with the appropriate software installed (Panlab, Barcelona, Spain), registering the rotations of the rat by 90° towards the side of the lesion (ipsilateral rotations) or to the side opposite to the lesion (contralateral rotations). From these measurements, the computer then counted the number of full (360°) turns in both directions at 10 min intervals. On the 13th day after the unilateral 6-OHDA-induced lesion, rats were tested for rotational behavior in response to APO. Immediately following the injection of this drug, after a 5 min acclimatization to the bowl conditions, ipsi- and contralateral rotations were automatically recorded for 60 min. In the case of treatment with L-DOPA and sildenafil, alone or in combination, the rotational behavior was measured immediately after the first and penultimate doses of the tested drugs for 120 or 160 min, respectively. Due to the negligible number of ipsilateral rotations, only contralateral rotations were analyzed in Section 2 and Section 3.

### 4.4. The Tissue Sample Collection

One hour after the last dose of L-DOPA and sildenafil, alone and in combination, rats were sacrificed by decapitation. This time point was chosen based on our previous study showing that the peak dopamine (DA) concentration in the STR and SN after i.p. administration of 12.5 or 25 mg/kg L-DOPA was 1 h [48]. The pharmacokinetics of sildenafil are not fully understood, including the information on the percentage of the drug that can cross the blood–brain barrier and the peak concentration time in the brain. However, it has been reported that an increase in cGMP concentration in the mouse midbrain was observed 30 min after intravenous administration of sildenafil at doses of 5 or 10 mg/kg [74]. Therefore, it can be assumed that in our study this effect could be slightly delayed after the i.p. administration of the used doses of sildenafil.

The brains were rapidly extracted from the skulls, and then the left- and right-sided STR and SN were quickly dissected on an ice-chilled glass plate. The procedure for dissecting these brain structures was as follows: First, the rat brains were divided into two parts, cutting them on the ventral side, perpendicular to the long axis of the midbrain, at the caudal border of the central eminence. This approach provided access to the left- and right-sided SN, which could be easily identified and dissected. Then, the anterior part of the brain was turned to the dorsal side and, after moving the cerebral cortex aside, the right and left STR was exposed and became accessible for dissection. The isolated tissue samples were stored at −80 °C until further procedures were applied.

### 4.5. Determination of the Concentrations of DA, 5-HT, and Their Metabolites in the Tissue Homogenates

The reverse-phase high performance liquid chromatography (HPLC) with the coulometric detection was used to assay tissue concentrations of DA, serotonin (5-HT), and their metabolites: 3,4-dihydroxyphenylacetic acid (DOPAC), homovanillic acid (HVA), and 5-hydroxyindoleacetic acid (5-HIAA) in the striatal and nigral homogenates, separately for the left (ipsilateral) and right (contralateral) side. The analysis was carried out as described previously [48,75].

Briefly, tissue samples were weighted, homogenized in ice-cold 0.1 M of perchloric acid containing 0.05 mM of ascorbic acid, and centrifuged (10,000× *g*) for 15 min. The supernatants were filtered using 0.2 µm cellulose filters (Alltech Associates Inc., Deerfeld, IL, USA) and then injected into the HPLC system which consisted of a P680 pump, an ASI-100 autosampler and a thermostated column compartment TCC-100 (Dionex, Germering, Germany) equipped with a Hypersil Gold C18 analytical column (150 × 3.0 mm i.d., 3 µm particle size) fitted with a 10 × 3 mm precolumn (Thermo Fisher Scientific Inc., Waltham, MA, USA). Detection was performed using a Coulochem III detector (ESA Inc., Chelmsford, MA, USA) equipped with a guard cell (ESA 5020, electrode potential set at 600 mV) and a dual electrochemical analytic cell (ESA 5010, applied potential E1 = −200 mV, E2 = 300 mV). Temperatures of the analytical cell as well as of the column were maintained at 30 °C. The mobile phase consisted of 35 mM of a citrate/47 mM disodium phosphate buffer (pH 4.2), supplemented with 0.25 mM of EDTA, 0.25 mM of 1-octanesulfonic acid sodium salt, 2.4% methanol and 1.3% acetonitrile. DA, 5-HT, and their metabolites were quantified by comparing the peak area of the tested samples with freshly prepared standards, run on the day of analysis. The approximated retention times of the measured substances were as follows: for DOPAC = 4.26; DA = 5.31, 5-HIAA = 7.98, HVA = 12.53, and 5-HT = 14.2 min. The obtained data were analyzed using a Chromeleon 6.8 software (Dionex, Germany).

### 4.6. Statistics

A statistical analysis of the behavioral data was performed in two steps. First, the number of contralateral rotations in 10 min intervals was calculated for each rat from a given group. Then, a two-way ANOVA for the repeated measures was conducted and followed (if significant) by a Newman–Keuls test for post hoc comparisons of the studied groups. Statistical analyses of the total number of contralateral rotations calculated for the 0–60 or 61–120 min periods between groups of rats treated chronically only with the vehicle, L-DOPA (12.5 mg/kg), and sildenafil (2 or 6 mg/kg; sil2 and sil6), and sil2+L-DOPA (12.5) or sil6+L-DOPA (12.5) combinations, were performed using a two-way ANOVA followed (if significant) by a Newman–Keuls test for post hoc comparisons of the studied groups.

The significance of differences in the concentrations of DA, 5-HT, and their metabolites between the examined groups was performed using a two-way ANOVA followed by a Newman–Keuls test. The total rate of DA catabolism was calculated from the ratio of the common DA metabolite HVA to DA concentration. It was expressed as the catabolism rate index (HVA/DA) × 100. The oxidative, MAO-dependent DA catabolism was calculated as the ratio of DOPAC to DA concentrations. It was presented as an index of (DOPAC/DA) × 100. The total rate of 5-HT catabolism was calculated from the concentration ratio of its metabolite 5-HIAA to 5-HT and was expressed as the catabolism rate index (5-HIAA/5-HT) × 100. All these indices were calculated using concentrations from individual tissue samples. The significance of differences in the concentrations of DA, DOPAC, and HVA between the ipsi- and contralateral side of the 6-OHDA group treated with the vehicle were analyzed by Student’s *t*-test for dependent samples.

*p* values of 0.05 or less were considered to indicate statistical significance. The statistical analysis was carried out using STATISTICA 10.0 Software (Statsoft, Inc., Tulsa, OK, USA).

Figures illustrating behavioral and biochemical data were created using GraphPad Prisma 9.1.2. Software (San Diego, CA, USA).

## Data Availability

The data that support the findings of this study are available from the corresponding author (lorenc@ifpan.krakow.pl) upon reasonable request.

## Supplementary Materials

The following supporting information can be downloaded at: https://www.mdpi.com/article/10.3390/molecules29184318/s1. Figure S1. Representative chromatograms for DA, 5-HT, and their metabolites in the homogenate of the ipsi- and contralateral striatal tissue sample as well as in the homogenate of the ipsi- and contralateral nigral tissue sample from unilaterally 6-OHDA-lesioned rat treated chronically i.p. with the vehicle.
