# Supplementary material for: The Effect of Chronic Treatment with the Inhibitor of Phosphodiesterase 5 (PDE5), Sildenafil, in Combination with L-DOPA on Asymmetric Behavior and Monoamine Catabolism in the Striatum and Substantia Nigra of Unilaterally 6-OHDA-Lesioned Rats
Source: Molecules. 2024 Sep 11;29(18):4318. doi: 10.3390/molecules29184318 (PMC11434559; doi:10.3390/molecules29184318)
Supplement: Supplementary file 1 [file molecules-29-04318-s001.zip › molecules-3144411-supplementary.pdf]

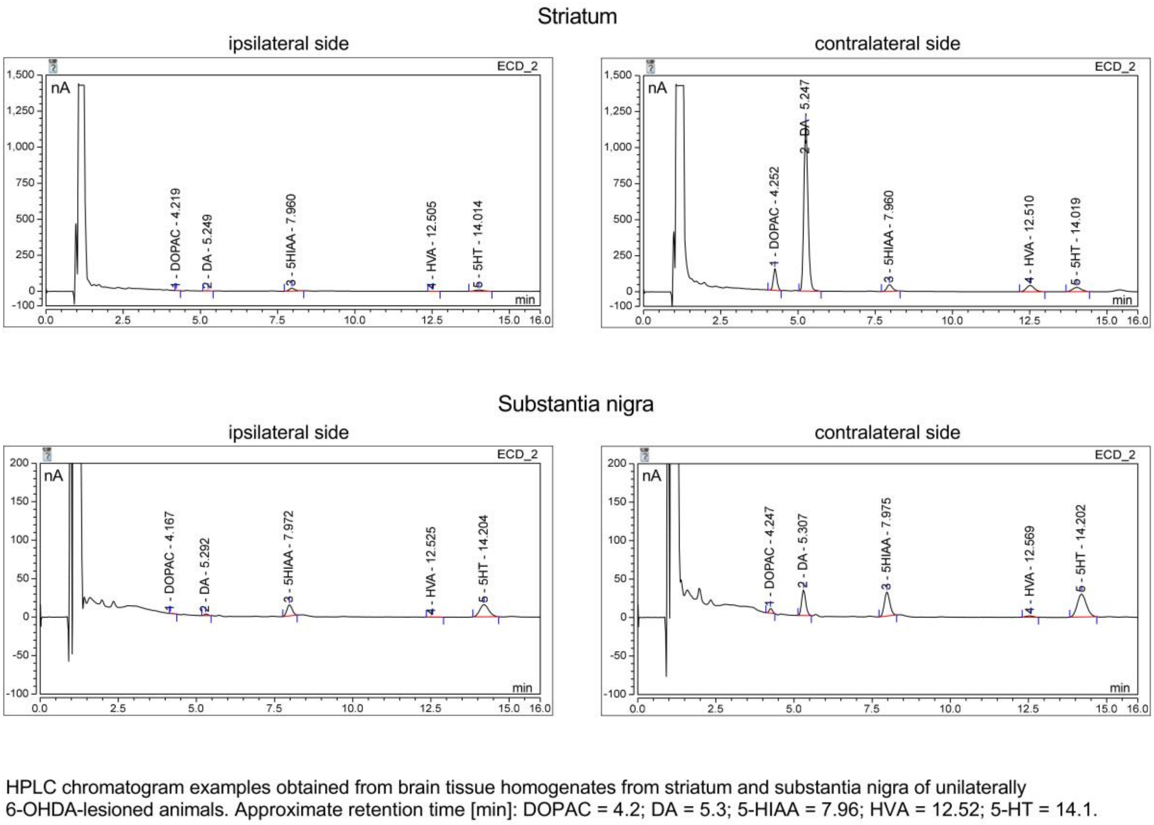

Figure S1. Representative chromatograms for DA, 5-HT, and their metabolites in the homogenate of the ipsi- and contralateral striatal tissue sample as well as in the homogenate of the ipsi- and contralateral nigral tissue sample from unilaterally 6-OHDA-lesioned rat treated chronically i.p. with the vehicle.
